# The SALOME study: recruitment experiences in a clinical trial offering injectable diacetylmorphine and hydromorphone for opioid dependency

**DOI:** 10.1186/1747-597X-10-3

**Published:** 2015-01-26

**Authors:** Eugenia Oviedo-Joekes, Kirsten Marchand, Kurt Lock, Scott MacDonald, Daphne Guh, Martin T Schechter

**Affiliations:** Centre for Health Evaluation & Outcome Sciences, Providence Health Care, St. Paul’s Hospital, 575- 1081 Burrard St., Vancouver, BC V6Z 1Y6 Canada; School of Population and Public Health, University of British Columbia, 2206 East Mall, Vancouver, BC V6T 1Z3 Canada; Providence Health Care, Providence Crosstown Clinic, 84 West Hastings Street, Vancouver, BC V6B 1G6 Canada

**Keywords:** Opioid dependency, Injectable diacetylmorphine, Injectable hydromorphone, Recruitment, Randomized clinical trial

## Abstract

**Background:**

The Study to Assess Long-term Opioid Medication Effectiveness (SALOME) is a two-stage phase III, single site (Vancouver, Canada), randomized, double blind controlled trial designed to test if hydromorphone is as effective as diacetylmorphine for the treatment of long-term illicit opioid injection. Recruiting participants for clinical trials continues to be a challenge in medical and addiction research, with many studies not being able to reach the planned sample size in a timely manner. The aim of this study is to describe the recruitment strategies in SALOME, which offered appealing treatments but had limited clinic capacity and no guaranteed post-trial continuation of the treatments.

**Methods:**

SALOME included chronic opioid-dependent, current illicit injection opioid users who had at least one previous episode of opioid maintenance treatment. Regulatory approvals were received in June 2011 and recruitment strategies were implemented over the next 5 months. Recruitment strategies included ongoing open communication with the community, a consistent and accessible team and participant-centered screening. All applicants completed a pre-screening checklist to assess prerequisites. Applicants meeting these prerequisites were later contacted to commence the screening process.

**Results:**

A total of 598 applications were received over the two-year recruitment period; 130 were received on the first day of recruitment. Of these applicants, 485 met prerequisites; however, many could not be found or were not reached before recruitment ended. For the 253 candidates who initiated the screening process, the average time lapse between application and screening date was 8.3 months (standard deviation [SD] = 4.44) and for the 202 randomized to the study, the average processing time from initial screen to randomization was 25.9 days (SD = 37.48; Median = 15.0).

**Conclusions:**

As in prior trials offering injectable diacetylmorphine within a supervised model, recruiting participants for this study took longer than planned. The recruitment challenges overcome in SALOME were due to the high number of applicants compared with the limited number that could be randomized and treated. Our study emphasizes the value of integrating these strategies into clinical addiction research to overcome study-specific barriers.

**Trial registration:**

ClinicalTrials.gov: NCT01447212.

## Background

Opioid dependency remains to be a major public health concern in North America and worldwide. Abstinence oriented treatment has shown to benefit small proportions of people struggling with opioid-dependence [1]; opioid substitution and maintenance treatment continues to be the best approach to stop the use of illicit opioids [2]. Oral methadone is the most used and studied of these treatments with demonstrated effectiveness at reducing illicit drug use and improving health and psychosocial outcomes [3]. Few other opioids besides oral methadone have been studied for the treatment of opioid dependency. Among these other opioids, injectable diacetylmorphine (i.e., pharmaceutical-grade heroin [DAM]), dispensed in a controlled and medically supervised setting, has shown to be effective in the treatment of long-term opioid injectors not sufficiently benefitting from oral methadone [4].

Despite that several randomized clinical trials (RCT) in Europe and Canada have demonstrated the effectiveness of DAM, very few countries have adopted it as part of their addiction treatment system. There is a clear need for alternative treatments as effective as injectable DAM that health care systems could adopt to attract and retain long-term street opioid injectors into treatment. One such alternative could be HDM [5] and the SALOME (Study to Assess Long-term Opioid Medication Effectiveness) study was designed to test this. SALOME is a randomized double blind controlled trial testing if injectable HDM (a licenced pain medication) is as effective as DAM for the treatment of long-term opioid-dependent individuals who are not benefitting sufficiently from conventional treatments, and if a transition to the oral equivalent of HDM and DAM after six-months is as effective as the injection form.

Recruiting participants for clinical trials can be slow and difficult, and much effort is dedicated to reach the required sample size within a reasonable timescale and budget [6–8]. For example, a review of RCTs across a wide array of medical conditions in the United Kingdom showed that nearly half of the trials received an extension of some kind due to the struggle to recruit the planned sample size [9]. Although it is unclear why some trials recruit more effectively than others, it has been suggested that studies providing treatments that are desired and/or only available through the trial, or studies comparing two similar treatments, might have fewer challenges with recruitment [10–13].

Methodological and empirical studies regarding recruitment in clinical trials for addiction are sparse. Some evidence suggests that RCTs in this field face similar challenges to those aforementioned in medical research [14]. However, addiction treatment trialists also encounter barriers specific to this field [15]. For example, motivation to participate may be more challenging when recruiting participants who have generally unstable daily living conditions and difficulties attending appointments [16]. Studies with similar populations have addressed the importance of building trust with the target population, support them to keep appointments, and provide ancillary services for other needs as part of their recruitment strategies [17]. Moreover, traditionally employed strategies for motivating participation, such as telephone reminders [18], may not be feasible with this population. These unique factors necessitate discussion amongst addictions researchers to better inform strategies tailored to benefit recruitment in forthcoming addiction trials.

Context specific challenges related to recruitment for RCTs with DAM treatment have also been documented. Every RCT testing DAM has had longer than expected recruitments and in some cases had to enrol fewer participants than planned [19]. A variety of context-specific reasons explain this. For example, in Spain, by the time the study was able to start enrolling participants, injection street heroin use had dropped drastically (heroin users were smoking it instead) and the sample size could not be reached [20]. In a Belgian study, one of the main reasons street heroin users did not apply was the concern over the limited length of access to the study medications [19]. In our prior study, NAOMI (North American Opiate Medication Initiative), the site in Vancouver received more than 1,000 applications; however, the study criteria did not reflect the realities of the intended sample population and most applicants did not even meet minimal requirements at first contact [21].

SALOME compared two treatments appealing to the target population that were only available through the study. Despite the advantages that this scenario presents for recruitment, experience in prior RCTs testing DAM suggested that offering desirable treatments might not be sufficient to meet the planned sample size within a reasonable time. The aim of the present study is to describe the recruitment experiences of the SALOME trial and discuss the strategies that were employed. This paper could inform the development of recruitment strategies for upcoming clinical trials in addictions research, a field that faces unique barriers.

## Methods

### Study design

SALOME is a two-stage phase III, single site (Vancouver), randomized, double blind controlled trial involving a total of 202 participants. The study population is defined as chronic, opioid-dependent, injection drug users who are currently injecting and who have attempted at least one previous episode of opioid maintenance treatment. Specific eligibility criteria included: a) minimum age of nineteen years; b) currently residing in the greater Vancouver area of British Columbia, Canada; c) current regular use of injectable opioids; d) at least two verified prior addiction treatment attempts, including one episode of opioid substitution treatment; e) at least five years or more of illicit opioid use; f) poor health or psychosocial functioning; and g) provision of fully informed consent. Participants were excluded if they did not meet inclusion criteria, had medical conditions contraindicated for treatment with injectable opioids or criminal justice involvement resulting in prolonged incarceration. The study was reviewed and received ethical approval from the University of British Columbia/Providence Health Care research ethics board.

Sample size requirements for the study were calculated based on illicit heroin use as the primary outcome. Using data derived from the results of our prior NAOMI trial, a non-inferiority design with an expected decline of 20 days (standard deviation = 11.0) of illicit heroin use from baseline, a margin of 4 days, a power of 0.90, an expected loss-to-follow-up rate of 0.05 and a one-sided alpha level of 0.05, requires 202 participants (101 per group) for phase I. With a sample size of n = 202, we can expect that approximately 172 participants will enter into phase II. Under the same assumptions as above, this will yield a non-inferiority trial with power = 0.86.

In phase I, half of the 202 participants were randomized to receive injectable DAM, and the other half to receive injectable HDM. In phase II, participants still receiving treatment in phase I were randomized to continue injection treatment exactly as in phase I on a blinded basis or to switch to the oral equivalent of the same medication (DAM or HDM).

DAM or HDM, either orally or injected, could only be prescribed and self-administered in the study clinic under supervision of health care providers, up to three times daily. Study treatments were provided for six months in each phase, followed by a one-month period during which participants still being treated with DAM or HDM were tapered and transitioned to other treatments available in the community (e.g., methadone). Upon entry into the trial, each participant had access to a defined range of primary care services matched to the prevalent conditions seen in injection drug users. A psychosocial support worker was also assigned who provided individual counselling services and case management (i.e., coordinated inter-disciplinary services such as housing and disability support).

### Clinic capacity and timeline

**Figure 1:**
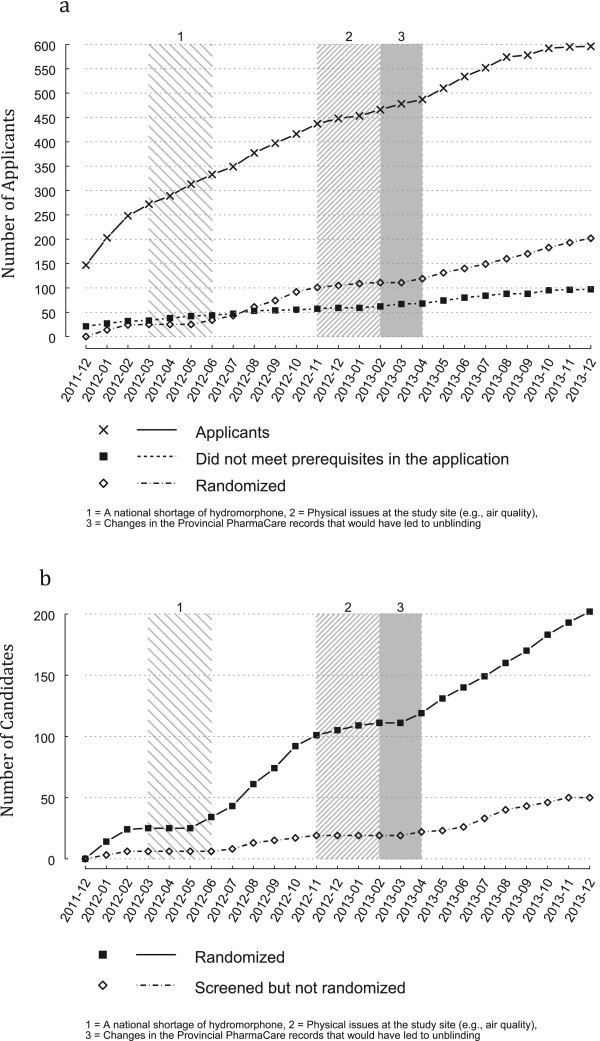
**Cumulative recruitment, screening and enrolment to the SALOME trial. a)** Cumulative recruitment into the SALOME trial. Number of applicants, ineligibles, and participants randomized to the SALOME trial over time. **b)** Cumulative screening of candidates in the SALOME trial. Number of candidates and randomized participants screened for the SALOME trial over time.

To be able to operate, the SALOME trial site requires an Exemption under Section 56 of the Canadian federal *Controlled Drugs and Substances Act*. This allows health care staff and researchers to use controlled narcotics for scientific purposes. Several security features are needed in a clinic in order to receive such an exemption and only one, the Vancouver clinic that was used in our prior NAOMI study, met these requirements at the time. The study clinic was planned to accommodate up to 95 participants receiving injectable medications. Once study participants completed the treatment or were switched to oral medications (phase II) or dropped out from the study, new participants could be screened and allocated into the study. The limited clinic capacity resulted in a varying enrolment pace of six to twenty participants per month, instead of four to five per week as planned.It later became clear that in order to meet the service needs of the participants, the clinic could best operate with no more than 85 participants receiving injectable medications per day. In addition to clinic capacity, three external events halted enrolment for five months. First, a national shortage of hydromorphone occurred four months into the study (Figure 1a) and caused a two month halt to recruitment while the research and clinical care teams developed a strategy and gained the necessary approvals for the study pharmacy to manufacture hydromorphone for participants only. Due to air quality issues requiring construction in the building where the study clinic is placed (an old bank), recruitment was halted for one month to reduce the physical demands on the clinic. Finally, changes in the Provincial PharmaNet recording of all drug prescription practices that would have led to the potential unblinding of clinic health care workers also caused a two month pause to recruitment while the clinical team worked with the authorities to develop a site specific protection feature. These impediments and the reduced clinic capacity delayed reaching the sample size by seven months approximately.

### Community engagement

Regulatory approvals were received in June 2011 and recruitment strategies were implemented over the next five months. A team was assembled and included the principal investigator (EOJ), research coordinators (KL; KM), the lead nurse, lead physician (SM), and communications director. This team engaged in recruitment collaboratively and remained consistent throughout the study. One of the Research Coordinators (KL), who had over a decade of experience working with the target population in the neighbourhood where the study was conducted, was fully devoted to overseeing recruitment.

Before recruitment opened, the team established a conveniently located research office, telephone recruitment number, email address, and study website. As part of efforts to reach every opioid user in Vancouver, we identified agencies, not-for-profit groups and health care providers that work with the target population and would be able to assist relaying information about the trial. These agencies were contacted and offered an opportunity to have the team provide an information session for staff and clients.

Over these months, we hosted approximately 20 formal information sessions with various agencies, including drug user groups (e.g., Vancouver Area Network of Drug Users, Drug Users Resource Centre), the research ethics board, health care teams (e.g., Vancouver Native Health, Downtown Eastside Women’s Center), social workers, legal agencies, health officers, and politicians. These sessions included a 20 to 30 minute verbal presentation of the study and time for questions. Common discussion topics included eligibility criteria, the duration of treatment and transition planning at the end of the study. Information packages were distributed either at the end of the session or in lieu of; materials included relevant publications from the NAOMI trial, contact information and answers to frequently asked questions. The team continued to provide updates and information sessions during the study as requested by these groups or as new information about the study became available.

During this time, a Community Advisory Board (CAB) was also established and is comprised of various stakeholders in the community. The purpose of the CAB is to provide guidance and feedback to the SALOME team with respect to accrual, retention, compliance, access, and ethical issues surrounding study. The CAB also serves as a link between the community and the SALOME team and has been a place for discussions of strategies for continuation of provision of study treatments.

These community engagement activities provided the team with an opportunity to learn of the perceived strengths and weaknesses of the trial and their implications for recruitment and the study itself. Strengths included the benefit of the medication and the need for alternative treatments. The lack of guaranteed continuation of study treatments was the main worry expressed by the community and patients. Also, some addictions physicians expressed concern over patients leaving successful treatments and binging in street drug in order to qualify for the study.

### Recruitment process

Since we experienced a combination of positive interest in SALOME as well as opposition, the team prepared the first day of recruitment (December 19, 2011 between 9am and 5pm) taking into account that the number of applicants could be high or low. In addition to opening the recruitment telephone number, recruitment tables were set up at three key agencies serving the target population. Information about this first day was displayed in advance at several local agencies and the CAB.

In order to filter minimal requirements without signing consent and engaging in full screening, applicants were interviewed at first contact with a pre-screening checklist, which assessed prerequisites for initiating screening and collected contact information. The recruitment telephone number was the primary method for applying to the study after the first day of recruitment. A chronological numbered list of applicants was created and used to determine their processing order. Once an applicant’s number was reached on this list, the applicant was re-contacted. Upon reaching the applicant, the on-file pre-screening checklist was reviewed and the applicant was asked if he or she was still interested in participating. If so, an appointment was made to begin the screening process. If an applicant could not be found, the chronological order of their application was not displaced. For example, candidates who were incarcerated when the team initially contacted them were able to reconnect and begin the screening process upon release.

Screening to the study required a minimum of three appointments, each lasting an hour or longer in duration. There were no costs to participants and at each stage in the screening process candidates were provided a modest stipend for their time amounting to $35 CAD over the three visits (average hourly wage in British Columbia is $10.50 CAD). During the first visit, candidates signed the informed consent, and the process of verifying eligibility criteria was started. At the second screening visit, candidates completed the baseline questionnaires. Candidates meeting criteria up to the end of the second screening visit were scheduled to meet with the study physician for a full medical examination and confirmation of all criteria. This visit was multi-disciplinary and patient centered; candidates were introduced to the health care providers and services available at the clinic (reception, nurses, physicians, psychosocial team) and a psychosocial assessment was completed. This process was effective for screening and providing an orientation to all aspects of the care available at the clinic.

Following the medical examination and confirmation of all criteria, the study physician notified the research team of participant eligibility. A member of the research team then randomized the participant using the trial randomization database. Randomization notifications were then distributed to the clinic coordinator and pharmacy manager for preparation of the participant’s treatment initiation.

### Analysis

Mean, standard deviation [SD] and proportions were used to describe time lapse, the flow of applications and meeting or not prerequisites or study criteria for applicants and candidates.

## Results

**Figure 2:**
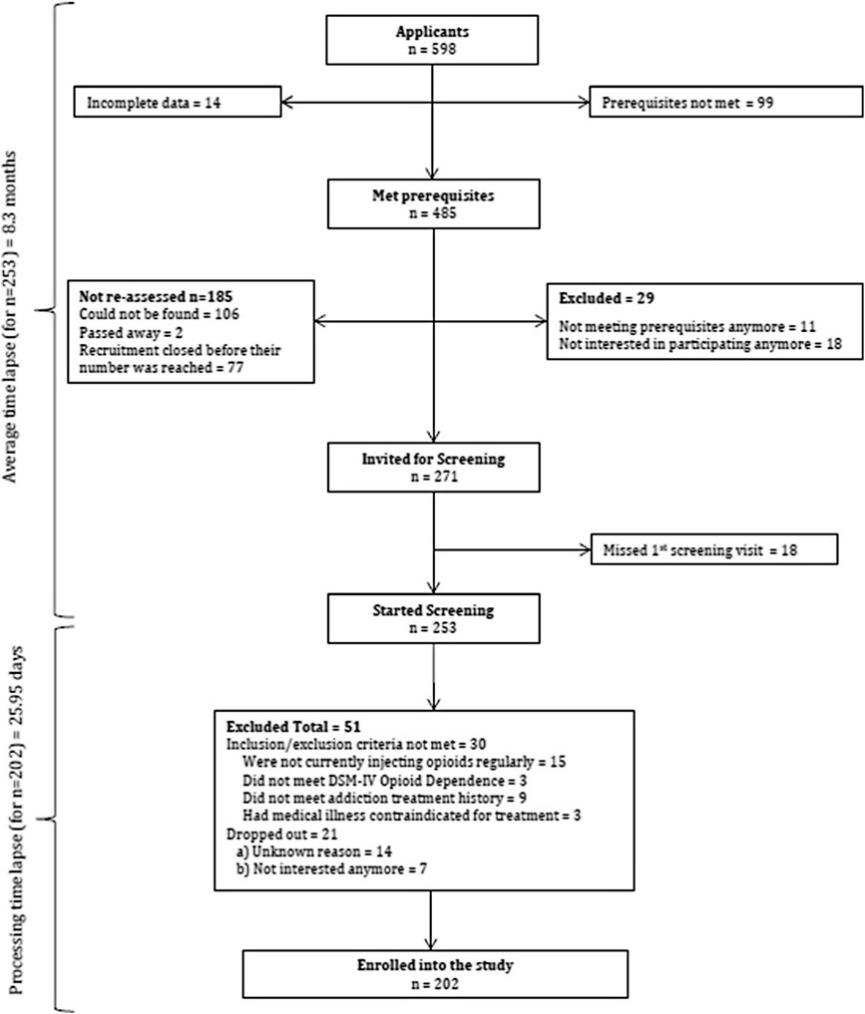
**SALOME screening flow chart.** Stages of screening in the SALOME trial and number of applicants at each stage.

From December 2011 to December 2013, a total of 598 (196 female, 397 male, 4 transgender-female and 1 with missing gender) applications were received for the SALOME trial. Figure 1a and 1b show the progress of the applications over the two-year period. On the first day 130 individuals applied to the study (Figure 1a). These applicants were randomly assigned a number that was used to determine their processing order. Individuals who applied after these 130 applicants were added to this list in chronological order. After the first day the number of applicants continued to increase steadily over the two-year period at a pace faster than the study had capacity to enrol. Therefore, efforts to actively promote recruitment (e.g., recruitment posters, recruitment tables) were halted.Figure 2 shows the flow of the applications through to randomization. From the total number of applicants, 485 (81.1% of total 598 who applied) met the prerequisites on the pre-screening checklist. Among those eligible for screening at this stage, 159 were female, 321 were male, 4 were transgender-female and one applicant’s gender was missing. For various reasons shown in Figure 2, we could not re-assess 185 of these 485 applicants (e.g., could not be found, passed away, etc.) and an additional 29 applicants were excluded for no longer meeting study prerequisites or no longer being interested in participating. The rate of applicants not meeting the prerequisites on the pre-screening checklist throughout the two-year recruitment period remained constant (Figure 1a).A total of 253 (52.2% of those who met prerequisites on the pre-screening checklist; 76 female, 174 male, 3 transgender-female) candidates signed the informed consent and initiated screening. Due to the limited clinic capacity and the external situations that halted recruitment, the average time lapse between the point of first application and first screening visit was 8.3 (SD = 4.44; Q1 = 6.33; median = 8.67; Q3 = 11.03) months. Their average age was 44.76 (SD = 9.61; Q1 = 38.0; median = 45.0; Q3 = 52.0). Thirty candidates (11.8% of those who started screening; 11 female, 19 male) were excluded due to not meeting at least one of the inclusion or exclusion criteria. In addition, 14 (5 female, 9 male) candidates started the screening process but did not continue for unknown reasons and 7 (1 female, 6 male) expressed that they were no longer interested in participating in the study. The primary reasons for exclusion of the 30 candidates was the regularity of current opioid injection (n = 15) and not sufficiently attempted OST or other types of addiction treatment (n = 9). For those eligible candidates, the average number of days between the first screening visit and randomization date was 25.95 (SD = 37.48; Q1 = 9.0; median = 15.0; Q3 = 23.0; Figure 1b). For 28 participants, the time lapse between first consent and randomization was more than 30 days, mainly because of challenges maintaining contact and rescheduling of missed appointments.

## Discussion

As in prior trials offering injectable DAM within a supervised model, recruiting participants for this study took seven months longer. However, this additional time can be attributed to the extraneous events and limited clinic capacity. Without these events and with a larger clinic, recruitment into the trial would have been even faster than expected.

The challenges that the research team had to overcome for recruitment into SALOME were due to the high number of applicants compared with the limited number that could be randomized and treated. Our recruitment plan was based on recommendations from recruitment into clinical trials in general, the addictions field and prior experiences of this research team [21]. The recruitment strategies that were instrumental in overcoming these challenges included the community engagement process [13, 22], the consistent, accessible and experienced team [13] and a participant-centered screening process [12, 14].

The community engagement process was highly valuable in forming partnerships with key agencies working with the study population and the patients. A very important long-term aim of the community engagement plan was to lay the foundation for future continuation of supervised injectable treatment. With these partnerships and their vested interest in this, we had the opportunity to establish this groundwork. In addition, the CAB was an important forum for communicating information about arising barriers as well as devising collaborative strategies for overcoming them. Throughout the study, there was open and ongoing communication between the research team and the community. For example, when a physician contacted any team member with concerns regarding a patient being included into the study, meetings were promptly held to discuss these concerns, while upholding mutual goals to protect participant/patient autonomy and the scientific integrity of the study.

The commitment of the community and stakeholders strengthened our ability to recruit the necessary number of participants despite the many external barriers affecting recruitment. In addition, having an experienced team improved communication and the consistency of research procedures. The team’s cohesion proved beneficial as the recruitment processes remained constant and the team’s skills matured over time as barriers continued to arise. Messages and information relayed about the study were also homogeneous through media, information materials and our team. Each message was tailored for the intended (i.e., participant, health care provider) audience, yet was consistent to minimize the negative impact arising barriers might pose to ongoing recruitment. For example, when we experienced the hydromorphone shortage, we quickly developed a plan for addressing this issue and a script for communication. This reduced uncertainty and maintained community engagement and support.

As in many RCTs, the stages of screening can be a burden on candidates and SALOME was no exception to this. In an effort to reduce the loss of candidates during the screening stages while maintaining scientific rigor, our procedures aimed to be participant-centered. Overall interactions with candidates were supportive and understanding of the daily struggles applicants had. Most candidates required additional support from the research team or other agencies to, for example, attend the multiple appointments required for screening. Sometimes a team member met candidates at their residence to accompany them to appointments. If the candidate had a social worker or advocate, the research team worked closely with that person to support the candidate throughout the process. The team understood this process as an opportunity to engage candidates not just in the study but also with the health care system, attending to their particular needs and barriers.

Precise estimates regarding how many applicants were needed to reach the planned sample size was difficult to achieve. For example, in many occasions we would have to go down the ordered applicant list because many consecutive applicants could not be found. Other times, they no longer met the prerequisites, were not interested or were doing well on other treatments. Due to this unpredictability, the research team continued to accept new applications until the final sample size was reached. This decision was made in collaboration with the clinical team and community advisory board. Like other trials [17], it was agreed that the opportunity to make contact with applicants, provide information about the trial and other treatments was favorable despite the possibility that recruitment might be complete before we could enrol them. In our communication with these applicants, they were informed of their application number, the number we were presently contacting and our best estimate of the time lapse or the likelihood of being contacted before reaching the sample size. Applicants’ desire to apply to the study despite this and the patience they demonstrated is indicative of their willingness to access alternative treatments for their long-term opioid dependency.

While our recruitment strategies were effective in reaching the target sample and allowing us to overcome extraneous challenges, a limitation of the data collected in this study is the impossibility to ascertain which strategy or combination of strategies was most influential or cost-effective. Given that our main strategy for recruitment was based on a strong community engagement plan, it is difficult to determine which of these activities contributed to the overall effectiveness of this strategy. For example, word of mouth was the primary response received when applicants were asked how they heard about the study. With such a response, we are not able to determine if the sample could have been reached, for example, by focusing efforts on information sessions to drug user groups only, without contacting health care workers. Also, the extensive time lapse caused by the external events makes comparison with refusal rates from other RCTs problematic. Nevertheless, it is important to note that approximately one-third of applicants meeting prerequisites were not invited to initiate screening because we were unable to find them or because recruitment ended before their number was reached. Since the pre-screening checklist data was provided prior to consent, the information collected was very limited and so we have little data about those we were unable to contact.

This study contributes to the building evidence [14, 15, 17, 19, 23] regarding the recruitment experiences and strategies used by addictions trialists who face barriers unique to this field. The recruitment strategy yielded the necessary sample size but also provided the foundation for constructive discussion regarding continuation of treatment. The strategies put in place in the SALOME trial and the positive outcomes emphasize the importance of the time investment in three particular areas. Well before the trial began, a core team was dedicated to establishing respectful relationships with the community, which created a supportive system in the face of unpredictable circumstances that affected the recruitment timeline. A consistent and committed recruitment team was dedicated and maintained through the study for this task. Finally, frontline research and clinical staff worked toward ensuring applicants had the support they needed to complete the screening process and were successfully allocated into the trial. Our experiences reflected in this manuscript could help other researchers in the planning and implementation of future RCTs in the addictions field with the potential for improving recruitment timelines and retention outcomes.

## Conclusions

SALOME is searching for evidence-based treatments that can attract and retain individuals struggling with long-term opioid dependency into treatment. Although we reached the target sample size, SALOME recruitment faced serious challenges specific to the study context. These challenges were successfully managed through open communication with the community, a consistent and accessible team and a participant-centered screening process. Our study emphasizes the value of integrating these strategies into clinical addiction research to overcome study-specific barriers.
